# Dietary Practices in Saudi Cerebral Palsy Children

**DOI:** 10.12669/pjms.314.7812

**Published:** 2015

**Authors:** Nouf S. Al-Hammad

**Affiliations:** Dr. Nouf S. AlHammad, BDS, MS Department of Pediatric Dentistry and Orthodontic, College of Dentistry, King Saud University, PO Box 60169 Riyadh 11545; Saudi Arabia

**Keywords:** Dietary practices, Cerebral palsy, Children

## Abstract

**Objectives:**

To determine the dietary practices of Saudi cerebral palsy (CP) children.

**Methods:**

A self-administered questionnaire was used to collect the following information from parents of CP children: demographics, main source of dietary information, frequency of main meals, foods/drinks used for main meals and in-between-meals.

**Results:**

Parents of 157 CP children participated. Parents were divided into three, while children were divided into two age groups. The main sources of dietary information included popular media (46.5%) and dentist (36.3%). Most of the children had three meals (71.3%) or two meals (24.8%) daily. Choices for main meals included meats (68.8%), vegetables (65.6%), fruits (28.4%) and puddings (38.9%). The main three drinks choices with main meals included packed juices (59.9%), bottled water (58.8%) and fresh fruit juices (33.1%). The choices for in-between meals snacks included biscuits (61.1%), potato chips (51.6%), fruits (43.9%) and chocolates (41.4%). The choice of drinks with snacks was similar to that used with main meals. In cross-tabulation, older parents used meat (p=.03) and soft drinks (p=.04) more often for their children’s main meals. Older children were given meat (p=.004) and soft drinks (p=.04) more often with main meals. Older children were given potato chips as snacks more often than younger children (p=.02), and there was a trend towards use of chocolates as snacks in older children (p=.08).

**Conclusion:**

Parents of CP children need to be educated about dietary practices of their children especially in areas such as the use of packed juices, dairy products, soft drinks and chocolates.

## INTRODUCTION

Cerebral palsy (CP) is a common form of neuromuscular disability^1^ and considered one of the most common handicapping conditions among Saudi children. Feeding difficulties are common in neurologically impaired children and impact negatively on food intake, growth, neurodevelopment and general health of these children.^1^,^2^ The North American “Growth in Cerebral Palsy Project” demonstrated a clear correlation between the degree of motor impairment and severity of feeding difficulties and reported feeding problems in majority of the cerebral palsy children they evaluated.^3^ Several feeding problems have been reported in cerebral palsy children such as need for help with feeding, frequent chocking, stressful and prolonged feeding, frequent vomiting and chewing and swallowing dysfunction.^2^,^4^

Cerebral palsy children have a reduced self-cleansing function of the oral cavity due to drooling and abnormal movement of the tongue and facial muscles; their diet is usually rich in pastry food and carbohydrates, and their caregivers have difficulty in practicing an appropriate oral hygiene.^5^,^6^

It is well known that a balanced diet also affects children’s dental health. Various factors may play a role in the ability of certain foods to cause dental disease. These include; the potential of the food to: produce acids that lower the salivary pH and provide an environment for bacterial growth and decalcification of enamel, adhere to the teeth, stimulate saliva production, buffer the production of acids and the rate at which a food dissolves.^7^ Dietary habits have been considered as strong predictor for the development of new dental caries.^8^,^9^

Although CP population in Saudi Arabia has been shown to exhibit a high prevalence of oral diseases in previous and more recent reports^10^-^13^, no study has been conducted on their dietary practices. Since dietary practices are usually modified in these children because of their disability, it is important to assess their dietary practices and evaluate them in relation to oral health. The purpose of this study was to determine the dietary practices of Saudi cerebral palsy children.

## METHODS

This questionnaire type study was conducted in the Disabled Children’s Association Center (DCAC) in Riyadh. The institutional and ethical approvals were obtained from King Saud University, College of Dentistry Research Center and DCAC Research Ethical Committee. A self-administered questionnaire was especially developed to collect the following information from the parents of Saudi CP children.

- Demographic information such as age and gender of parents and their CP children.
- Main source of oral health information for their children.
- Frequency of main meals per day.
- Foods/drinks used for main meals.
- Foods/drinks used between-meals.

The questionnaire was pre-tested on parents not participating in the study. Some changes were made to make it more comprehensible for parents.

After obtaining a written consent from the parents; the questionnaires were handed over to the parents at the time of their CP children routine dental care in the DCAC dental clinic. All the questionnaires had a covering letter explaining the research objectives and ensuring anonymity/confidentiality of the information obtained. Non Saudi parents and parents of children with disabilities other than CP were not included in the study. Parents who could not read or write were assisted in completion of the questionnaire.

Collected data were entered into a computer and analyzed using Statistical Package for Social Sciences “SPSS” (Version #16).

Descriptive statistics, including the means and standard deviations, as well as frequencies, were calculated. Chi-square and Fisher’s Exact tests were used to determine any significant difference (P≤0.05) in responses in terms of parental age group, parental gender, CP children’s age group and gender.

## RESULTS

Parents of 157 Saudi CP children registered in the center completed the questionnaire. Mostly mothers (86.6%) completed the questionnaire; mean parental age was 34.0 years (SD 7.3, Range 20-58). Mean age of CP children was 6.7 years (SD 2.7, Range 2-12) [Male 57.7%, Female 42.3%]. For the purpose of analyses; the parents were divided into three age groups; 20 - 30 years, 31 - 40 years and 41 - 58 years. Similarly, children were divided into two age groups, 2 - 6 years and 7 - 12 years (Table-I). Of the 157 CP children, 153(97.5%) were spastic and 4(2.5%) had ataxia. As for the type of physical disability; 6 (3.8%) children were hemiplegic, 68 (43.3%) had deplagia, 10 (6.4%) paraplegia, 50 (31.8%) quadriplegia and 23 (14.7%) had non-specific physical disabilities. Comparison of the responses by CP type and physical disability were not carried out due to uneven distribution.

**Table-I:** Age and gender distribution of the parents and their CP children.

Participants	Children		Parents		
Age (Years)	2-6	7-12	20-30	31-40	41-58
Number (%)	79 (50.3)	78 (49.7)	63(40.1)	68 (43.3)	26 (16.6)
Gender	Males	Females	Males	females	
Number (%)	90 (57.7)	67 (42.3)	21 (13.4)	136 (86.6)	
Total	157		157		

The main sources of dietary information for the parents of CP children included popular media [television, radio, newspapers] (46.50%), dentists (36.30%), child’s health care center (8.30%), child’s school (4.50%) and others (4.50%). Most of the children had either three meals (71.3%) or two meals (24.8%) per day. Choices for main meals included meats (68.8%), vegetables (65.6%), fruits (48.4%) and puddings (38.9%). The main three drinks choices with main meals included packed juices (59.9%), bottled water (58.8%) and fresh fruit juices (33.1%). The choices for in-between meals snacks included biscuits (61.1%), potato chips (51.6%), fruits (43.9%) and chocolates (41.4%). Cheese consumption during snacking was reported by about one-third of the participants only. The choice of drinks with snacks was similar to that used with main meals (Table-II).

**Table-II:** Food and drinks for main meals and snacks of the CP children.

Main Meals		Snacks	
Foods (%)	Drinks (%)	Foods (%)	Drinks (%)
Meats; 68.8	Packed Juices; 59.9	Biscuits; 61.1	Packed Juices; 66.9
Vegetables; 65.6	Bottled Water; 58.0	Potato Chips; 51.6	Bottled Water; 58.0
Fruits; 48.4	Fresh fruit juices; 33.1	Fruits; 43.9	Fresh Fruit Juices; 36.3
Puddings; 38.9	Soft Drinks; 9.6	Chocolates; 41.4	Soft Drinks; 7.6
Others; 19.7	Tap Water; 4.5	Cheese; 35.7	Tap Water; 3.2
	Flavored fizzy drinks; 5.7	Others; 5.7	Flavored Fizzy drinks; 3.8
	Others; 12.1		Others; 6.4

Cross-tabulation between parental age groups and various dependent variables showed that older parents as compared to younger parents used meat more often for their CP children’s main meals (*p*=.03); and used more soft drinks (*p*=.04) and less fresh fruit juices (*P*=.05) with main meals. Older children were given meat more often as main meal compared to younger children (*p*=.004); and were also given soft drinks with main meals more often (*p*=.04). Older CP children were given potato chips as snacks more often than younger children (*p*=.02) and less other drinks such as milk with snacks (*p*=.05). There was an increasing trend towards use of chocolates as snacks in older children (*p*=.08). All other cross-tabulations were non-significant.

## DISCUSSION

Assessing dietary practices is a useful tool in identifying those children at high risk of oral diseases. Feeding difficulties in CP children usually lead to dietary inadequacies,^14^-^16^ which has been listed as one of the causative factors of gingival diseases.^17^ The present study has indicated that the dietary practices of the study population are generally satisfactory in terms of number of main meals per day and the type of food selected for the main meals; as it included most of the important food groups “meats, carbohydrates, vegetables and fruits”. The only deficient part was dairy products, as only few parents added dairy products to the main meals, under the category of others, as compared to rice, bread and pasta which were added more frequently. This might affect the calcium level in the body which is important nutrients for oral and general health.

Most of the studies conducted previously regarding CP children dietary intake evaluated the nutritional status, feeding difficulties and feeding methods in relation to growth and general health outcomes,^1^,^14^-^16^,^18^,^19^ but not in relation to their oral health. Packed fruit juices contain high level of sugars and have been reported as a risk factor for dental caries development.^20^ In the present study; the most commonly consumed drinks during main meals and snacking were packed fruit juices (59% & 66.9% respectively). This could be attributed to the parental belief that packed fruit juices are not cariogenic. Carbonated drinks also have high sugar content and are potentially cariogenic. Their role in the etiology of erosion and dental caries is well known.^21^-^23^ In the studied group of CP children, consumption of soft drinks during main meals or snacking was not high (9.6% & 7.6% respectively), however, it was noticed that older parents used more soft drinks with main meals than younger parents and older children were given soft drinks with main meals more often. This could be explained by the fact that older children have the ability to select or ask for their drink of choice more than younger children and younger parents may be more aware of their negative effects.

Type and frequency of snacking are important factors in development of oral diseases.^24^ Mannaa and co-workers (2013)^24^ noticed a trend toward higher caries experience in relation to a higher snacking frequency. De Camargo and Antunes (2008)^25^ also reported that the intake of sweetened foods and beverages was the most relevant covariate associated with the prevalence of untreated caries in CP children. Some foods while not obviously cariogenic contain high level of sugar and fermentable carbohydrates; these foods include potato chips, biscuits and fruits. In the present study the choices for in between meals snacks included most commonly biscuits, potato chips, fruits and chocolates. Older children were given potato chips as a snack more often than younger children, and also a trend towards use of chocolate as a snack. This could be related to the older children’s role in the selection of their snack preferences or to the ability of the older children to eat potato chips.

Improved nutrition is linked to improved health outcomes and quality of life.^1^ In the absence of disease-specific dietary recommendations for use in individuals with CP, standard recommendations for dietary intakes of vitamins, minerals and trace elements should be utilized.^26^ Lifelong eating patterns and food habits are established during childhood, the parents of CP children need to be educated about dietary practices in their children. Sugar containing snacking should be discouraged and sweets should be limited to meal time only. Many food products not thought of as containing sugar do contain sugar. Therefore, educating parents about the type of foods that should be avoided and teaching them how to read and interpret labels on food are extremely important and will have a positive effect on the oral and general health of their children. A comprehensive dietary guidance should be developed by the health and education authorities and provided to the parents of CP children through their main sources of oral health information such as popular media and their dentists. More studies are needed to assess the dietary practices of cerebral palsy children in relation to their oral health in various parts of the world.

## CONCLUSION

The parents of CP children need to be educated about dietary practices of their children especially in areas such as the use of packed juices, dairy products, soft drinks and chocolates.
